# Management of angle-closure glaucoma with X-linked retinoschisis: a case report

**DOI:** 10.1186/s12886-023-02903-7

**Published:** 2023-04-17

**Authors:** Yanxia Li, Jia Li, Xingzhu Pan, Zhaoying Zhang, Yajuan Zheng

**Affiliations:** Department of Ophthalmology, The Second Hospital of Jilin University, Jilin University, 218 Ziqiang Street, Changchun, 130041 Jilin China

**Keywords:** X-linked retinoschisis, Angle-closure glaucoma, Malignant glaucoma, Case report

## Abstract

**Background:**

X-linked retinoschisis (XLRS), due to mutations in the *RS1* gene, is a common genetically determined form of macular degeneration. This report describes an unusual case of angle-closure glaucoma (ACG) with XLRS and discusses the treatment.

**Case presentation:**

A 39-year-old Chinese man with an X chromosome-recessive inherited c.489G > A variant in the *RS1* gene was diagnosed as XLRS and ACG, presenting with cystic macular lesions, shallow anterior chamber depth (ACD), and angle-closure with uncontrolled intraocular pressure (IOP). Malignant glaucoma occurred following trabeculectomy combining phacoemulsification with intraocular lens (IOL) implantation and goniosynechialysis. Subsequent anterior vitrectomy and irido-zonulo-hyaloid-vitrectomy (IZHV) effectively lowered IOP and deepened ACD, but the cystic cavity became larger.

**Conclusions:**

There is a potential risk of malignant glaucoma in ACG patients with XLRS after filtering surgery. Although anterior vitrectomy can effectively resolve aqueous misdirection, the macular retinoschisis may get worse. Awareness of this risk may aid in surgical planning and postoperative management in these patients.

## Background

X-linked retinoschisis (XLRS), which is characterized by macular retinoschisis with or without peripheral retinoschisis, is a hereditary retinopathy in males resulting in significant vision deterioration [1]. Young angle-closure glaucoma (ACG) has been described previously in patients with XLRS [2–4]. Furthermore, compared to older patients, younger ACG patients develop aqueous misdirection more frequently after trabeculectomy [5, 6]. It is a challenge for glaucoma surgeons to manage these young ACG patients with uncontrolled intraocular pressure (IOP) and a high incidence of surgical complications. Here we report an unusual ACG patient with XLRS in whom malignant glaucoma occurred and retinoschisis fluctuated during the treatment process.

## Case presentation

A 39-year-old man presented with progressive blurred vision and ocular pain in the left eye for the preceding 8 months, and the left eye had undergone laser peripheral iridectomy previously. His right eye underwent combined phacoemulsification, intraocular lens (IOL) implantation, goniosynechialysis, anterior vitrectomy, and irido-zonulo-hyaloid-vitrectomy (IZHV) to resolve malignant glaucoma after trabeculectomy 5 years ago. The patient denied a family history of eye diseases or a history of ocular trauma or systemic diseases.

**Fig. 1:**
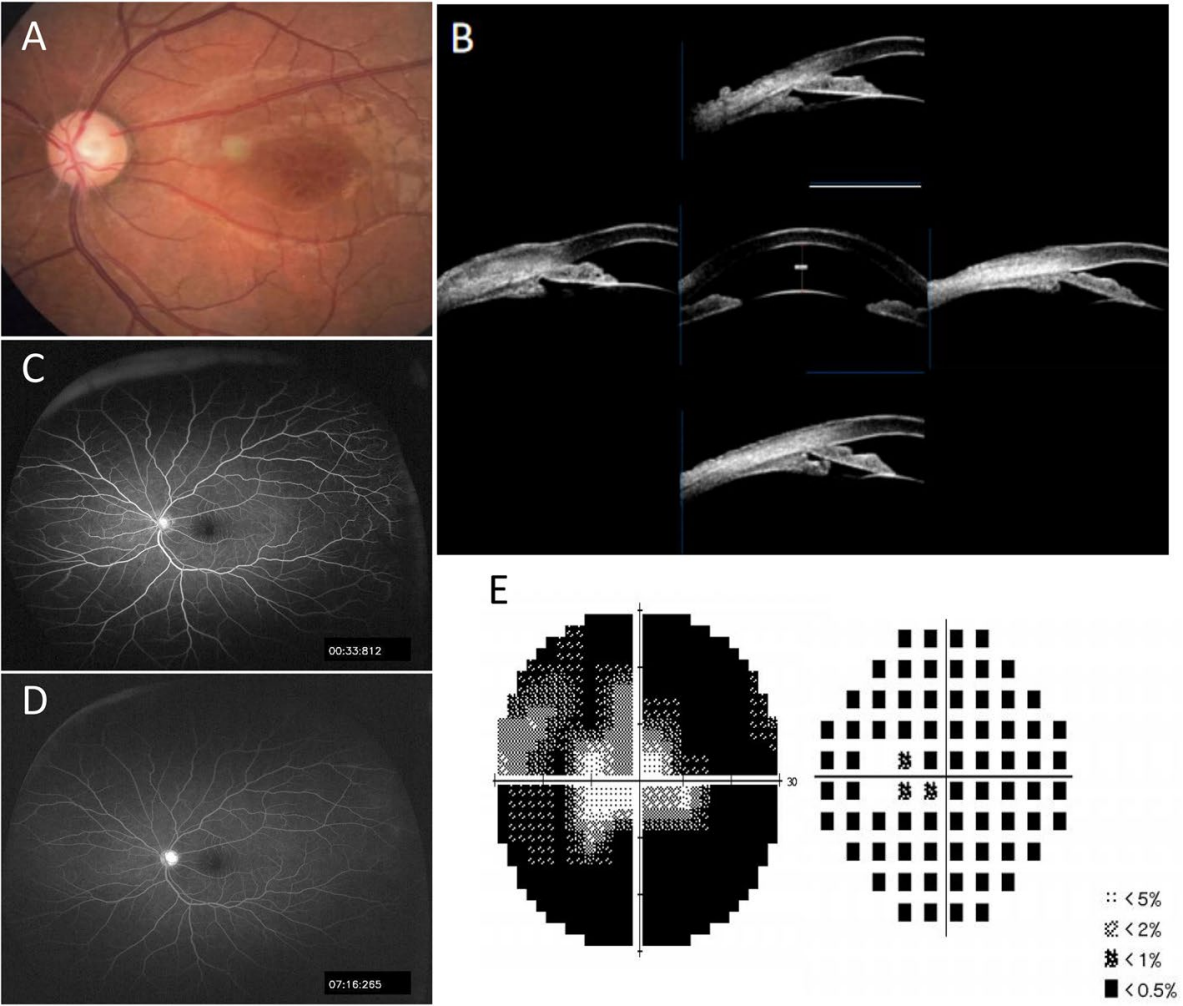
Ophthalmic examination of the left eye. A spoke-wheel pattern in the macular area was shown in the fundus photograph (**A**). UBM confirmed the forward rotation of the ciliary body and closed angle (**B**). A significant visual field defect (**E**) and normal fundus fluorescein angiography (**C**, **D**)

**Fig. 2:**
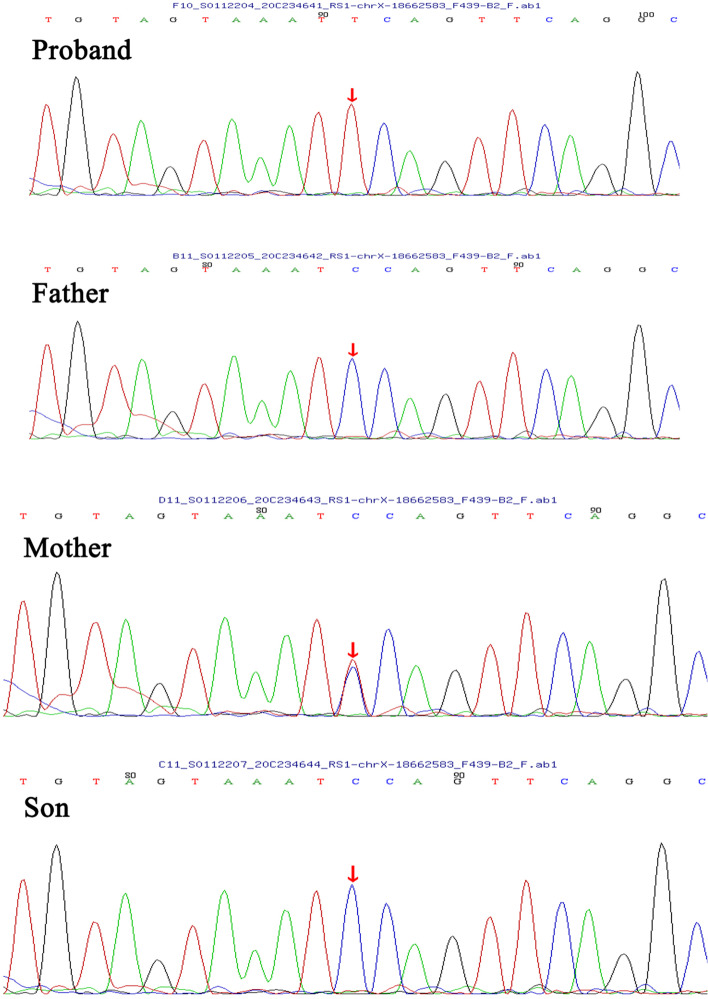
Partial electropherograms of the *RS1* exon 5. The proband was hemizygous for the c.489G > A variant, and his mother was heterozygous for the same variant. The wild-type sequence was detected in his father and son

**Fig. 3:**
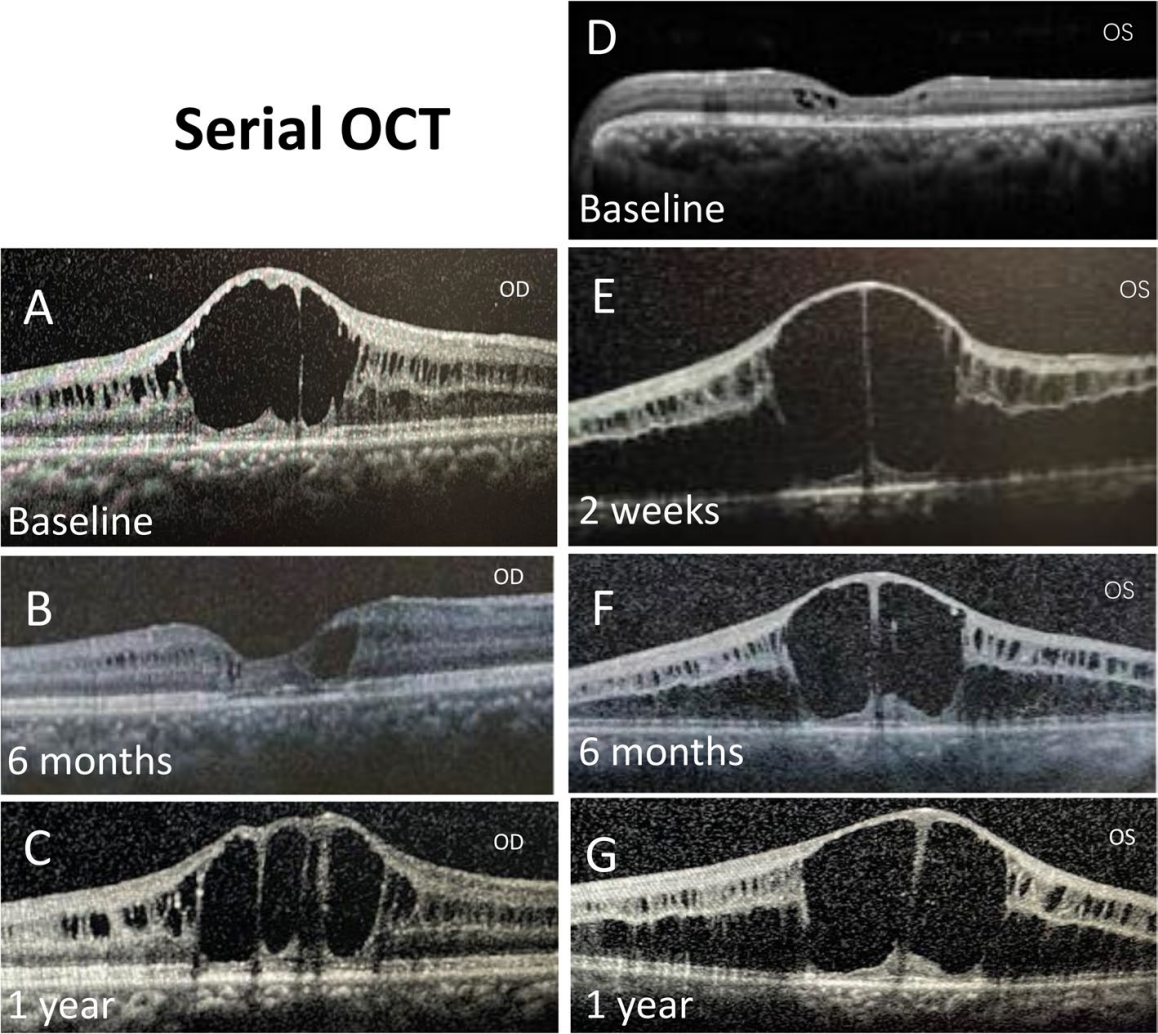
Serial OCTs of the central macula. Compared with preoperation (**A**), the foveal cyst in the right eye resolved at 6 months (**B**) after Nd: YAG laser posterior capsulotomy and get worse again at 1 year (**C**). A tiny cystic macular lesion showed in the left eye preoperatively (**D**), and the cystic cavity became larger after anterior vitrectomy at 2 weeks, 6 months and 1 year (**E**, **F**, **G**)

The best corrected visual acuity (BCVA) was 0.1 in the right eye (OD) and 0.4 in the left eye (OS), and the refractive state was hypermetropic (OD + 2.00/−1.00 × 120, OS + 2.5.00/−1.25 × 25). IOP measurements were 11 mmHg OD and 45 mmHg OS. The fundus of the right eye cannot be seen clearly because of posterior capsular opacity. Positive findings on slit-lamp examination of the left eye revealed mild corneal edema, clear lens and shallow central and peripheral anterior chamber. Fundus examination of the left eye was notable for a spoke-wheel pattern in the macular area and a pale optic disc (Fig. 1A). Gonioscopy demonstrated a synechial angle closure in both eyes. Ultrasound biomicroscopy (UBM) images of the left eye showed a shallow anterior chamber, closed angle, and ciliary body anteposition and forward rotation (Fig. 1B). The anterior chamber depth (ACD) was 1.55 mm and the axial length was 21.45 mm in the left eye measured by IOL Master. Perimetry revealed a significant visual field defect (Fig. 1E) and the result of fundus fluorescein angiography was normal (Fig. 1C and D). Macular optical coherence tomography (OCT) showed a tiny cystic macular lesion (Fig. 3D). The patient’s whole-exome sequencing revealed a hemizygous nonsense mutation, c.489G > A (p.W163X) in exon 5 of the *RS1* gene. Genetic testing was also detected in other family members, and the heterozygous for the same variant in the *RS1* gene was confirmed in his mother, while no variant in the *RS1* gene was detected in his father or his son (Fig. 2).

Carteolol, brimonidine tartrate, brinzolamide eye drops and oral acetazolamide were used to lower the patient’ IOP. Given the severe visual field damage and the elevated IOP on maximum anti-glaucoma medications, the left eye underwent trabeculectomy combined with clear lens extraction, IOL implantation and goniosynechialysis. On postoperative day 1, the eye showed a shallow central and peripheral ACD with a raised IOP of 35 mmHg. Malignant glaucoma was diagnosed, and anterior vitrectomy combined with IZHV was performed to balance the pressure between the anterior and posterior chamber. Following surgery, his IOP decreased to 11 mmHg and the ACD deepened.

After 2 weeks, the right eye underwent Nd: YAG laser posterior capsulotomy and bilateral OCT was performed. OCT showed that the schitic cavities in the left eye were worse than that before surgery (Fig. 3E) and lasted for 1 year (Fig. 3F and G). Interestingly, his foveal cyst in the right eye resolved at 6 months and get worse again at 1 year following Nd: YAG laser posterior capsulotomy (Fig. 3A, B and C). One year following treatment, his BCVA was 0.4 OD and 0.2 OS. The ACD remained stable at around 3.29 mm and IOP was between 12 and 14 mmHg in both eyes without anti-glaucoma drops.

## Discussion and conclusions

XLRS is associated with mutations in the *RS1* gene, which codes for retinoschisin, a secreted protein containing a discoidin domain [1]. Retinoschisin plays a critical role in cellular fluid balance, cellular adhesion, and cell-cell interactions across bipolar–photoreceptor synapses. Cystoid changes involved in various retinal layers can be caused by the absence of retinoschisin [1]. It was reported that congenital retinoschisis is one of the etiologies for young ACG patients [5]. Here we report an XLRS patient with ACG. In previous reports, the onset age of ACG, which mainly affects the elderly, was earlier in the setting of XLRS, and even some adolescents had suffered from ACG [2–4]. Further, the structures of short axial length and shallow ACD were demonstrated in these patients. Kellner et al. [7] also found that the refraction was hyperopia in most XLRS patients. These findings suggested that the early onset of ACG in XLRS patients may be due to anatomical structures such as shallow ACD and short axial length caused by the *RS1* gene mutation.

Although similar cases have been reported previously, this case had some unusual clinical course in that the macular cystic cavity of retinoschisis fluctuated during the treatment of glaucoma. In general, the natural history of XLRS displays long-term stability in most eyes [8]. In this patient, OCT of the central macula demonstrated marked fluctuations in the schitic cavities following surgery. Accumulated evidence showed that vitreous traction plays an important role in the development of foveal retinoschisis [9]. Therefore, we speculated that the worse macular retinoschisis was caused by vitreous traction following anterior vitrectomy. Furthermore, the local inflammation caused by the surgery may be another reason for the worse macular retinoschisis. Interestingly, we also found a transient resolution of macular retinoschisis after Nd: YAG laser posterior capsulotomy in the right eye. The high energy of the Nd: YAG laser can rupture the anterior hyaloid face and result in vitreous liquefaction immediately [10]. Hence, the most plausible explanation is that vitreous traction was temporarily decreased because of the vitreous liquefaction following Nd: YAG laser posterior capsulotomy process. With the dynamic change of the vitreous for a long time, the effect of releasing the vitreous traction by posterior capsulotomy disappeared.

This case is unique also in its reminder significance of the high incidence of malignant glaucoma after filtering surgery for ACG patients with XLRS. In this patient, aqueous misdirection developed in both eyes after trabeculectomy probably because of young age, short axial length, and rotation of the ciliary body. In addition, retinoschisin can regulate fluid balance by binding to the NaK ATPase [1]. One can speculate that another possible factor of post-operative malignant glaucoma may be the loss of functional retinoschisin causing fluid accumulation in the extracellular environment resulting in posterior pressure. Similarly, Huang et al. [4] also reported that an XLRS patient with ACG developed shallow ACD and uncontrolled IOP after trabeculectomy. Therefore, we should be alerted to the occurrence of malignant glaucoma in XLRS patients with ACG.

Topical carbonic anhydrase inhibitors are beneficial in cases of XLRS with ACG, reducing IOP levels and particularly causing significant resolution in foveal retinoschisis. Selvan et al. [3] reported that the IOP decreased with a significant collapse of the schitic cavities after a combination of topical dorzolamide and timolol maleate in an 11-year-old boy found to have XLRS with ACG. In addition, for such patients, clear lens extraction is a reasonable surgical option [2, 4]. If postoperative malignant glaucoma occurs, low-dose diode laser transscleral cyclophotocoagulation (TCP) might be worth trying. TCP could resolve malignant glaucoma by restoring the ciliary body orientation and deepening ACD [6]. Anterior vitreous liquefaction induced by post-TCP inflammation may also decrease vitreous permeability and promote the flow of aqueous humor through the vitreous cavity [6]. What’s more, vitreous traction decreased in the setting of liquefication may resolve macular retinoschisis [6].

In the case presented here, we considered that the lower IOP was needed for the patient to prevent optic nerve damage further. Thus, trabeculectomy combined with clear lens extraction was performed to maintain lower IOP instead of clear lens extraction alone in the first surgery. However, aqueous misdirection syndrome developed following surgery. On the other hand, due to a lack of sufficient understanding of ACG patients with XLRS at that time, we performed the anterior vitrectomy, a routine surgical option for malignant glaucoma, to resolve aqueous misdirection instead of TCP, resulting in worse macular retinoschisis. Therefore, this case warns us that we should carefully select the treatment methods for ACG patients with XLRS in future clinical work.

In conclusion, XLRS may be one of the etiologies for young ACG patients, and young ACG patients should undergo a fundus examination to exclude undiagnosed inherited retinal dystrophies. The present case demonstrates that malignant glaucoma occurs frequently in ACG with XLRS. Although anterior vitrectomy can effectively treat malignant glaucoma, the macular retinoschisis may get worse. It is a challenge for ophthalmologists to manage young ACG with XLRS.

## Data Availability

All data generated and analyzed during this study are included in this article.

## Abbreviations

XLRS: X-linked retinoschisis
ACG: Angle-closure glaucoma
ACD: Anterior chamber depth
IOP: Intraocular pressure
IOL: Intraocular lens
IZHV: Irido-zonulo-hyaloid-vitrectomy
BCVA: Best corrected visual acuity
OD: Right eye
OS: Left eye
UBM: Ultrasound biomicroscopy
OCT: Optical coherence tomography
TCP: Transscleral cyclophotocoagulation
